# Analogosedation during flexible bronchoscopy using a combination of midazolam, propofol and fentanyl – A retrospective analysis

**DOI:** 10.1371/journal.pone.0175394

**Published:** 2017-04-12

**Authors:** Tobias Müller, Kristina Thümmel, Christian G. Cornelissen, Stefan Krüger, Michael Dreher

**Affiliations:** 1 Division of Pneumology, University Hospital RWTH, Aachen, Germany; 2 Department of Pneumology, University Hospital, Freiburg, Germany; 3 Department of Pneumology, Florence-Nightingale-Hospital, Düsseldorf, Germany; Liverpool School of Tropical Medicine, UNITED KINGDOM

## Abstract

**Background:**

According to current guidelines flexible bronchoscopy is usually performed under sedation. Previously it has been demonstrated that combined sedation with e. g. the combination of midazolam and propofol or an opioid might have several advantages over sedation with just one sedative drug. However, little is known about the efficacy and safety of combined sedation with midazolam, fentanyl and propofol (MFP) compared to sedation with midazolam and fentanyl (MF) or midazolam and propofol (MP).

**Methods:**

We carried out a retrospective analysis of bronchoscopies performed under triple (MFP) or double sedation (MF and MP) in an academic hospital. 1392 procedures were analyzed (MFP: n = 824; MF: n = 272; MP: n = 296). In particular, we compared the occurrence of complications and the dosage of administered sedative drugs between the groups.

**Results:**

The occurrence of adverse events (MFP vs. MF: odds ratio (OR) 1.116 [95% CI 0.7741 to 1.604]; MFP vs. MP: OR 0.8296 [95% CI 0.5939 to 1.16] and severe adverse events (MFP vs. MF: OR 1.581 [95% CI 0.5594 to 4.336]; MFP vs. MP: OR 3.47 [95% CI 0.908 to 15.15]; all p>0.05) was similar in all groups. The dosage of midazolam was lower in the MFP compared to the MF or MP group (MFP vs. MF: Cohen’s d 0.075; MFP vs. MP: Cohen’s d 0.225; all p<0.001). In addition patients in the MFP group received significantly less propofol compared to the MP group (Cohen’s d 1.22; p<0.001).

**Conclusions:**

In summary we were able to demonstrate that triple sedation can safely be administered during flexible bronchoscopy and is associated with a reduced dosage of midazolam and propofol.

## Introduction

Flexible bronchoscopy is a well-established procedure for the diagnostic work-up of patients with a variety of respiratory diseases. In order to facilitate the procedure and to increase patient tolerance, comfort, and cooperation, current guidelines recommend offering sedation to all patients undergoing flexible bronchoscopy in the absence of contraindications [1,2].

Currently, there is no clear recommendation favoring one sedation regimen over any other. Nevertheless, the combination of the short-acting benzodiazepine midazolam and an opiate has been shown to be safe even in patients with pre-existing respiratory failure [3]. In this regimen the sedative and amnestic properties of benzodiazepines are combined with the analgesic and antitussive properties of opiates [3,4]. However, there is a significant variation in individual dose requirements and in the metabolism of midazolam, potentially leading to a prolonged recovery period. Therefore propofol has also been introduced for flexible bronchoscopy. The non-inferiority of propofol sedation versus combined sedation with midazolam and hydrocodone has been demonstrated in a randomized controlled trial [5]. Additionally, combined sedation with propofol and low dose midazolam during endoscopic procedures has been found to be safe and to be associated with a reduction in the amount of administered propofol compared to sedation with propofol alone [6,7]. This might be an advantage especially for patients suffering from cardiovascular disorders as cardiovascular depression and hypotension are a common side effect of propofol [8]. In accordance, patients receiving the combination of propofol and hydrocodone require less propofol and have a better cough suppression than those receiving propofol alone [9].

In summary there is good evidence that combined sedation during flexible bronchoscopy using two different drugs is safe and has several advantages compared to sedation with just one drug. However, much less is known about the safety of combined sedation with midazolam, an opioid and propofol. Such a triple sedation regime which has been used in a recent study could possibly combine the advantages of all three drugs (sedation, amnesia, analgesia, cough suppression, rapid onset of action and fast recovery) and allow to further reduce the propofol dosage [10]. On the other hand the application of three different drugs for sedation could also increase complications, including respiratory depression or cardio-circulatory events. Therefore, the aim of this retrospective analysis was to analyze the complication rate during flexible bronchoscopy with a sedation regime consisting of midazolam, fentanyl and propofol (triple sedation) compared to sedation with a regime consisting of only two different drugs (midazolam / fentanyl or midazolam / propofol).

## Material and methods

Data analysis was done with regard to the Declaration of Helsinki. The Institutional Review Board for Human Studies at RWTH (“Rheinisch-Westfälische Technische Hochschule”) University confirmed that a formal approval was not required as this retrospective analysis required neither an intervention nor irregularity of privacy or anonymity (EK 310/15).

An analysis of all flexible bronchoscopies between January 2012 and June 2016 performed in the Division of Pneumology at the University Hospital RWTH Aachen under moderate sedation was conducted. Bronchoscopies of patients with critical illnesses (e. g. patients with acute respiratory failure or patients needing vasopressor support) were performed on intensive care or intermediate care wards and were not included in the analysis. Furthermore if deep sedation was necessary, e. g. for staging of mediastinal lymph nodes via EBUS-TBNA or if a patient’s co-morbidities had been judged as severe by the attending physician rigid bronchoscopy was performed in the attendance of an anesthesiologist. These procedures were also not included in the analysis. All bronchoscopies were performed by a board certified specialist in pulmonary or internal medicine experienced in bronchoscopy or under their direct supervision. All physicians performing bronchoscopy were trained and experienced in airway management (endotracheal intubation by direct laryngoscopy and by fiberoptic bronchoscopy), as well as in the management of acute emergency situations (e. g. shock, cardiac arrhythmias, or acute respiratory failure) and critical care medicine. In case of complications a second physician experienced in bronchoscopy, as well as an emergency team from the ICU were available on short notice. Standard monitoring included electrocardiogram, oxygen saturation (SpO_2_), and non-invasive blood pressure (NIBP).

Original data was retrieved from an electronic patient record system (medico, Siemens, Germany). Only bronchoscopies performed under double (midazolam / fentanyl or midazolam / propofol) or triple (midazolam / fentanyl / propofol) sedation were analyzed. Procedures in which patients had been intubated under maintenance of spontaneous breathing (e. g. to allow cryobiopsies to be performed or to facilitate EBUS-TBNA) were excluded from the analysis, as endotracheal intubation requires a deeper level of sedation.

Demographic (age, sex) and epidemiological data (known cardio-vascular or chronic pulmonary co-morbidities), as well as the indication for bronchoscopy and—if available—data from pulmonary function tests (FEV_1_) were recorded and collected in a Microsoft Access database (Microsoft, Redmond, USA). Patients suffering from hypertension, chronic heart failure, ischemic heart disease, valvular heart disease, cardiac arrhythmias, pulmonary hypertension were categorized as having chronic heart disease, whereas patients suffering from asthma, chronic obstructive pulmonary disease (COPD), interstitial lung diseases or a FEV_1_ below 70% predicted were categorized as having a chronic pulmonary disease. Furthermore, the amount of administered sedative drugs (midazolam, fentanyl and propofol), the kind of intervention performed (e. g. broncho-alveolar lavage, endobronchial or transbronchial biopsy) and the occurrence of complications during the procedure documented by the investigator were recorded. The patient record system was also searched for complications which had not been documented in the bronchoscopy report, e. g. post-interventional pneumothorax occurring 24 h after transbronchial biopsy.

Complications were categorized in adverse events (AEs) and severe adverse events (SAEs) [11]. SAEs were defined as death within 24h after bronchoscopy, pneumothorax, major bleeding (defined as necessity for intubation or placement of a bronchus blocker), need for post-interventional ventilation (invasive or non-invasive), epileptic seizure or any event leading to an intensive or intermediate care unit admission after the procedure. AEs were defined as transient respiratory deterioration resolving under chin lift and jaw thrust maneuver or by increasing the flow rate of supplemental oxygen, need for oropharyngeal or endotracheal airway insertion which could be removed immediately after the procedure, short time mechanical ventilation during the procedure, hypotension, prolonged recovery after bronchoscopy as judged by the bronchoscopist, minor bleeding, or any event judged as complication in the bronchoscopy report not fulfilling the definition of a severe adverse event. However since the dosage of sedative drugs was another endpoint of this study, difficulties with sedation e. g. due to coughing were not considered as an AE even when this was documented by the bronchoscopist.

The occurrence of complications and the use of sedative drugs under sedation with midazolam, fentanyl and propofol (MFP) were compared to sedation with midazolam/fentanyl (MF) and to sedation with midazolam/propofol (MP). Additionally the MF and the MP group were combined (double sedation) and compared to the MFP group (triple sedation).

### Statistical analysis

Statistical analysis was performed using GraphPadPrism (GraphPad Software, La Jolla, USA). Unless otherwise stated, all data are presented as mean ± standard deviation (SD) after testing for normal distribution (Kolmogorov-Smirnov test). A two-group comparison was performed using the unpaired t-test for normally distributed data. For more than two groups ANOVA followed by Tukey’s multiple comparison test was used. For non-normally distributed data, the Wilcoxon signed rank test or the Kruskal-Wallis test was used and the interquartile range is given. The Fisher’s exact test was used for categorical data. Statistical significance was defined as a p value <0.05.

## Results

**Fig 1:**
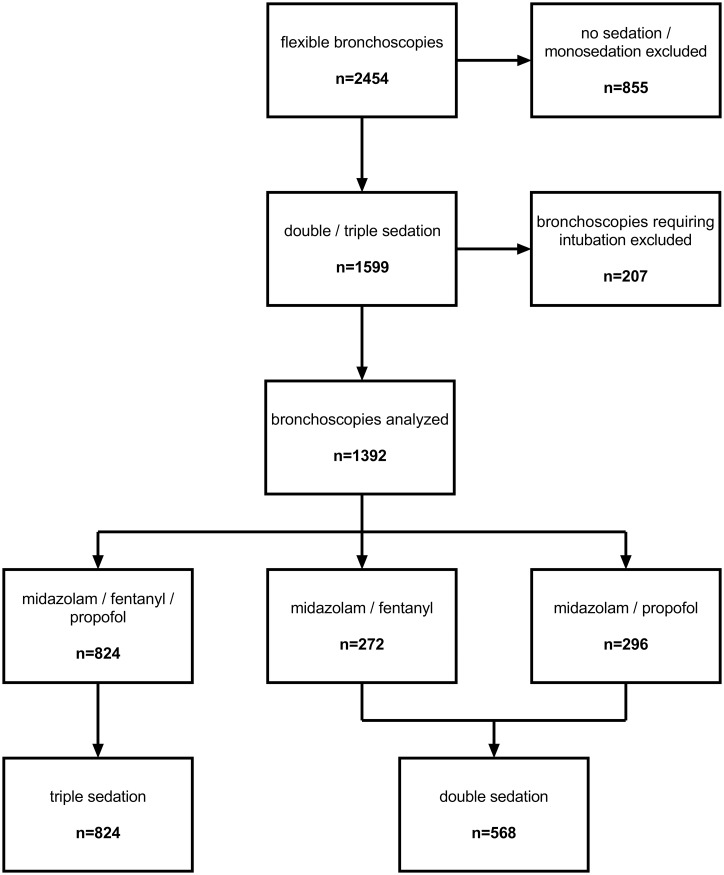
Patient flow chart.

A total of 1599 flexible bronchoscopies under double or triple sedation were performed in the bronchoscopy unit of the university hospital RWTH Aachen within the observed time period. Procedures performed under planned fiberoptic intubation were excluded from the analysis (n = 207) as these bronchoscopies had been almost exclusively performed under triple sedation (182 out of 207). The remaining bronchoscopies (n = 1392) were divided into three groups depending on the sedation regime: sedation with midazolam and propofol (MP; n = 296), sedation with midazolam and fentanyl (MF; n = 272), or sedation with midazolam, fentanyl and propofol (MFP; n = 824) (Fig 1).

### Patient characteristics

Detailed patient characteristics are displayed in Table 1. There were no differences between the 3 groups in terms of sex, body mass index (BMI) or pulmonary function test (FEV_1_). In contrast, patients in the MF group were older (Δ -5.708 years; 95% confidence interval [CI]: -8.087 to -3.329 years; p<0.0001) and patients in the MP group were younger compared to the MFP group (Δ 2.728 years; 95% CI: 0.4225 to 5.033 years; p = 0.0154). However, there was no difference in terms of age between the MFP and the combined MF/MP group (Δ 1.312; 95% CI: -0.2422 to 2.8660 years; p = 0.0980). Compared to the MFP group (51.6%) the prevalence of cardiovascular disorders was significantly higher in the MF (66.5%; p<0.0001 compared to MFP), and the combined MF/MP group (60.6%; p = 0.001 compared to MFP) In contrast the prevalence of structural lung disease was similarly distributed between the 3 groups (all p>0.05).

**Table 1:** Patient characteristics.

	MFP (n = 824)	MF (n = 272)	p^1^	MP (n = 296)	p^2^	MF+MP (n = 568)	p^3^
Male, n (%)	554 (67.2)	198 (72.8)	0.0973^4^	203 (68.6)	0.7175^4^	401 (70.6)	0.1962^4^
age, years	60.30±15.22	66.01±12.21	<0.0001^5^	57.57±14.38	0.0154^5^	61.61±14.03	0.0980^6^
BMI (kg/m^2^)	25.68±5.321^$^	25.53±4.36^#^	0.9499^5^	26.52±5.28	0.1385^5^	26.08±0.27^§^	0.2738^6^
cardio-vascular disease, n (%)	431 (52.3)	181 (66.5)	<0.0001^4^	163 (55.1^)^	0.4163^4^	344 (60.6)	0.0025^4^
chronic pulmonary disease, n (%)	382 (46.4)	129 (47.4)	0.7794^4^	135 (45.6)	0.8387^4^	264 (46.5)	>0.9999^4^
FEV1 (% predicted)	71.51±23.33^$^	68.81±20.68^#^	0.3938^5^	75.13±20.91^§^	0.1440^5^	72.29±21.01	0.6230^6^

Data are presented as number of patients (%) or mean ± standard deviation

^1^MFP vs. MF

^2^MFP vs. MP

^3^MFP (triple sedation) vs. MP and MF combined (double sedation)

^4^Fisher’s exact test

^5^ANOVA, followed by Tukey’s multiple comparison test

^6^unpaired t-test

data were available for:

^$^495 out of 824 patients (60.1%)

^#^152 out of 272 patients (55.9%)

^§^186 out of 296 patients (62.8%)

M = midazolam; P = propofol; F = fentanyl; FEV_1_ = forced expiratory volume in 1 second.

### Indication for bronchoscopy and interventions

The underlying diseases are summarized in Table 2; the diagnostic procedures performed are shown in detail in Table 3. Briefly, the number of diagnostic interventions was not different between the MFP and the MF group (Δ 0.03237; 95% CI: -0,08947 to 0,1542; p = 0.8073). However, more diagnostic interventions were performed in the MP compared to the MFP group (Δ -0,336; 95% CI: -0,4539 to -0,218; p<0.0001) due to more bronchoscopies with endo- and transbronchial biopsies, brush cytology and conventional transbronchial needle aspiration. The number of diagnostic interventions was also significantly higher in the MF/MP compared to the MFP group (Δ -0.1596; 95% CI: -0.07924 to -0.2399; p = 0.0001).

**Table 2:** Indications for bronchoscopy.

	MFP (n = 824)	MF (n = 272)	MP (n = 296)	MF+MP (n = 568)
lung cancer	252 (30.6)	74 (27.2)	93 (31.4)	167 (29.4)
pulmonary infection	179 (21.7)	45 (16.5)	29 (9.8)	74 (13.0)
interstitial lung disease	41 (5.0)	9 (3.3)	17 (5.7)	26 (4.6)
unexplained lymphadenopathy	7 (0.8)	1 (0.4)	13 (4.4)	14 (2.5)
unexplained pulmonary opacities	76 (9.2)	42 (15.4)	45 (15.2)	87 (15.3)
hemoptysis	70 (8.5)	29 (10.7)	26 (8.8)	55 (9.7)
other	199 (24.2)	72 (26.5)	73 (24.7)	145 (25.5)

Data are number of patients (%).

M = midazolam; P = propofol; F = fentanyl.

**Table 3:** Diagnostic interventions.

	MFP (n = 824)	MF (n = 272)	p^1^	MP (n = 296)	p^2^	MF+MP (n = 568)	p^3^
BAL	349 (42.4)	139 (51.1)	0.0137^4^	119 (40.2)	0.5369^4^	258 (45.4)	0.2715^4^
endobronchial biopsy	165 (20.0)	38 (14.0)	0.0305^4^	111 (37.5)	<0.0001^4^	149 (26.2)	0.0074^4^
transbronchial biopsy	83 (10.1)	18 (6.6)	0.0916^4^	47 (15.9)	0.0108^4^	65 (11.4)	0.4268^4^
brush cytology	46 (5.6)	16 (5.9)	0.8798^4^	46 (15.5)	<0.0001^4^	62 (10.9)	0.0003^4^
EBUS-TBNA	20 (2.4)	4 (1.5)	0.4754^4^	9 (3.0)	0.5297^4^	13 (2.3)	>0.9999^4^
conventional TBNA	11 (1.3)	0 (0)	0.0751^4^	10 (3.4)	0.0418^4^	10 (1.8)	0.5130^4^
interventions per procedure	0.8228±0.6867	0.7904±0.6344	0.8073^5^	1.159±0.9523	<0.0001^5^	0.9824±0.8355	0.0001^6^
**number of interventions**							
inspection only	271 (32.9)	86 (31.6)	0.7099^4^	78 (26.4)	0.0405^4^	164 (28.9)	0.1261^4^
1	437 (53.0)	160 (58.8)	0.1063^4^	127 (42.9)	0.0029^4^	287 (50.5)	0.3825^4^
2 or more	116 (14.1)	26 (9.6)	0.0606^4^	91 (30.7)	<0.0001^4^	117 (20.6)	0.0017^4^

Data are presented as number of patients (%) or mean ± standard deviation

^1^MFP vs. MF

^2^MFP vs. MP

^3^MFP (triple sedation) vs. MP and MF combined (double sedation)

^4^Fisher’s exact test

^5^ANOVA, followed by Tukey’s multiple comparison test

^6^unpaired t-test

BAL = bronchoalveolar lavage; M = midazolam; P = propofol; F = fentanyl.

### Medication

**Fig 2:**
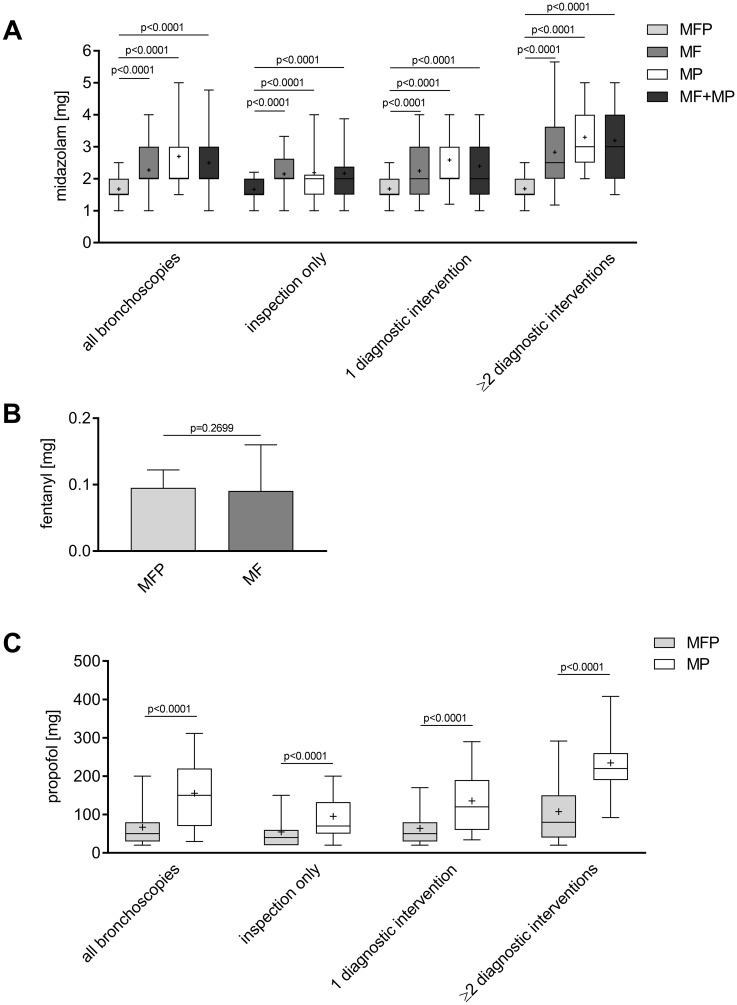
Consumption of sedative drugs. (A) The median midazolam dosage in the MFP, the MF, the MP and the combined MF/MP group is given. Boxes represent the interquartile range, whiskers report maximum and minimum. (B) Mean ± SD of administered fentanyl in the MFP and MF group is given. (C) The median propofol dosage in the MFP and the MP group is given. Boxes represent the interquartile range, whiskers report maximum and minimum. Means are indicated by +.

The median dosage of midazolam was significantly lower in the MFP group (1.5 mg [IQR 1.5 to 2.0 mg]) compared to the MF (2.0 mg [IQR 2.0 to 3.0 mg]; p<0.0001 compared to MFP], the MP (2.0 mg [IQR 2.0 to 3.0 mg]; p<0.0001 compared to MFP) or the combined MF/MP group (2.0 mg [IQR 2.0 to 3.0 mg]; p<0.0001 compared to MFP). As more diagnostic interventions were performed in the MP and MF/MP group compared to the MFP group, the amount of administered midazolam was also analyzed in bronchoscopies with similar complexity (inspection only, 1 diagnostic intervention, 2 diagnostic interventions or more). Nevertheless, even after adjusting for diagnostic interventions midazolam dosage was still lower in the MFP compared to all other groups (Fig 2A; S1 Table).

The amount of administered fentanyl was similar in the MF and the MFP group and did not differ significantly (Δ -0.004764 ± 0.00431; 95% CI: -0.01325 to 0.003718; p = 0.2699) (Fig 2B; S1 Table).

As shown in Fig 2C, the median dosage of propofol was significantly lower in the MFP compared to the MP group (MFP: 50 mg [IQR 30 to 80 mg] vs. MP: 150 mg [IQR 70 to 220 mg]; p<0.0001). In addition there was still a significant difference between the two groups when bronchoscopies were adjusted for diagnostic interventions (all p<0.0001). For individual patient data see S1 Table.

### Complications

Details of adverse events (AEs) and severe adverse events (SAEs) are listed in Table 4. The proportion of interventions with AEs in the MFP group (17.7%) was not different from the MF (16.2%; p = 0.5810 compared to MFP), the MP (20.6%; p = 0.2948 compared to MFP) or the MF/MP group (18.5%; p = 0.7233 compared to MFP). Among AEs minor bleedings followed by transient respiratory deterioration were the most common events. Other AEs were rare. Transient respiratory deterioration occurred more often in the MFP compared to the MF group (7.5% vs. 3.3% [p = 0.0150]).

**Table 4:** Complications.

	MFP (n = 824)	MF (n = 272)	p^1^	MP (n = 296)	p^2^	MF+MP (n = 568)	p^3^
**AEs**	146 (17.7)	44 (16.2)	0.5810^4^	61 (20.6)	0.2948^4^	105 (18.5)	0.7233^4^
minor bleedings	77 (9.3)	30 (11.0)	0.4114^4^	33 (11.1)	0.3645^4^	63 (11.1)	0.3186^4^
transient respiratory deteroriation	62 (7.5)	9 (3.3)	0.0150^4^	28 (9.5)	0.3186^4^	37 (6.5)	0.5248^4^
short time mechanical ventilation	6 (0.7)	0 (0.0)	0.3458^4^	1 (0.3)	0.6828^4^	1 (0.2)	0.2514^4^
insertion of oropharyngeal / endotracheal tube	9 (1.1)	0 (0.0)	0.1227^4^	0 (0.0)	0.1226^4^	0 (0.0)	0.0131^4^
hypotension	2 (0.2)	3 (1.1)	0.1011^4^	3 (1.0)	0.1187^4^	6 (1.1)	0.0693^4^
arrhythmia	4 (0.5)	0 (0.0)	0.5774^4^	4 (1.4)	0.2188^4^	4 (0.7)	0.7227^4^
prolonged recovery period	5 (0.6)	2 (0.7)	0.6860^4^	0 (0.0)	0.3335^4^	2 (0.4)	0.7073^4^
interruption of the procedure	2 (0.2)	1 (0.4)	0.5754^4^	1 (0.3)	>0.9999^4^	2 (0.4)	>0.9999^4^
other	10 (1.2)	2 (0.7)	0.7405^4^	2 (0.7)	0.7422^4^	4 (0.7)	0.4219^4^
**AEs depending on diagnostic interventions**							
inspection only	39(14.4)	7 (8.1)	0.3414^4^	12 (15.4)	0.5732^4^	19 (11.6)	0.8830^4^
1 diagnostic intervention	77 (17.6)	30 (18.8)	0.7499^4^	30 (23.6)	0.1567^4^	60 (20.9)	0.2866^4^
2 or more diagnostic interventions	30 (25.9)	7 (26.9)	>0.9999^4^	19 (20.9)	0.4162^4^	26 (22.2)	0.5426^4^
**SAEs**	19 (2.3)	4 (1.5)	0.4758^4^	2 (0.7)	0.0838^4^	6 (1.1)	0.1012^4^
pneumothorax	10 (1.2)	1 (0.4)	0.3097^4^	0 (0.0)	0.0711^4^	1 (0.2)	0.0331^4^
ICU / IMC admission	5 (0.6)	3 (1.1)	0.4178^4^	1 (0.3)	>0.9999^4^	4 (0.7)	>0.9999^4^
need for mechanical ventilation	2 (0.2)	0 (0.0)	>0.9999^4^	1 (0.3)	>0.9999^4^	1 (0.2)	>0.9999^4^
severe bleeding	4 (0.5)	2 (0.5)	0.6418^4^	1 (0.3)	>0.9999^4^	3 (0.5)	>0.9999^4^
seizure	2 (0.2)	0 (0.0)	>0.9999^4^	0 (0.0)	>0.9999^4^	0 (0.0)	0.5166^4^
death	0 (0.0)	0 (0.0)	>0.9999^4^	1 (0.3)	0.2643^4^	1 (0.2)	0.4080^4^

Data are presented as number of patients (%)

^1^MFP vs. MF

^2^MFP vs. MP

^3^MFP (triple sedation) vs. MP and MF combined (double sedation)

^4^Fisher’s exact test

M = midazolam; P = propofol; F = fentanyl.

Because the MP and MF/MP group were not entirely comparable to the MFP group in terms of diagnostic interventions, the occurrence of complications was also analyzed depending on the number of diagnostic interventions (inspection only, 1 diagnostic intervention, 2 diagnostic interventions or more). However, even after adjusting for diagnostic interventions there were still no significant differences in terms of adverse events between the MFP, the MF, the MP or the MF/MP group.

The occurrence of severe adverse events (SAEs) tended to be higher in the MFP compared to the other groups which was exclusively due to a higher incidence of pneumothorax in the MFP group (MFP: 1.2%; MF: 0.4% [p = 0.3097 compared to MFP]; MP: 0.0% [p = 0.0711 compared to MFP]; MF/MP: 0.2% [p = 0.0331 compared to MFP]). Other SAEs apart from pneumothorax were rare and did not differ between the groups.

## Discussion

Previous studies have been able to demonstrate that sedation during flexible bronchoscopy using two different drugs, e. g. midazolam combined with an opioid or propofol, is safe and might have several advantages over sedation with just one drug [3,4,9]. To the best of our knowledge, the present study compared for the first time in a large patient cohort double sedation with either midazolam/fentanyl (MF) or midazolam/propofol (MP) to triple sedation with midazolam, fentanyl and propofol (MFP). Thereby we could show that patients in the MFP group received less midazolam compared to the other groups and less propofol compared to the MP group. These findings are in line with previous studies demonstrating that the dosage of sedatives can be reduced if combined sedation is used [3,6,7,9]. Whereas the difference in the amount of midazolam was small and most likely not clinically relevant, the reduction in the propofol dosage was considerable and might be favorable for patients suffering from cardiovascular diseases [8]. In contrast, there were no differences in the amount of administered fentanyl between the MFP and the MF group. A likely explanation is that opioids are usually given at a fixed dose at the beginning of the intervention whereas the dosage of propofol or midazolam is usually titrated until a sufficient level of sedation is achieved [3].

Overall, the complication rate in all groups was within the expected range and a similar proportion of bronchoscopies with an AE was observed in the MFP, the MF, the MP and the MF/MP group [5,9,11]. In accordance a recent study performing bronchoscopy under sedation with midazolam, hydrocodone and propofol reported also a low complication rate [10]. The distribution of the different complications classified as an AE was slightly different between the groups, e. g. transient respiratory deterioration was reported more often in the MFP and MP compared to the MF group, most likely due to the use of propofol, as hypoxemia (oxygen saturation below 90%) has been reported as a common complication in bronchoscopies under propofol sedation [11]. However, respiratory deterioration rarely required mechanical ventilation, or oropharyngeal / endotracheal tube insertion during the procedure and post-interventional mechanical ventilation was needed even less frequently. More diagnostic interventions were performed in the MP and MF/MP compared to the MFP group suggesting a higher likelihood of complications in these groups. Though, even when this was taken into account there were no significant differences in the occurrence of AEs between the groups. It is also necessary to mention that the prevalence of cardiovascular diseases was higher in the MF and MF/MP compared to the MFP group though cardiovascular complications such as hypotension or cardiac arrhythmias were rarely observed and were not different between the groups.

The incidence of SAEs was very low and there was no statistically significant difference between the groups though there was a trend towards more SAEs in the MFP group exclusively due to a higher pneumothorax rate in the MFP group most likely not related to the sedation strategy.

This study has several limitations that need to be noted. Firstly, as the data were collected retrospectively we had to rely on the bronchoscopy report and on patient records to determine the occurrence of AEs and SAEs. Therefore the complication rate might have been under-reported in our study. Secondly, for the same reason data about patient comfort or operating conditions during bronchoscopy are lacking. Thirdly, no data are available on which purpose a distinct sedation regime (MFP, MF or MP) was chosen by the bronchoscopist. Some patients might have even been switched from MF or MP to MFP during the procedure, e. g. due to excessive coughing of the patient potentially leading to bias.

In conclusion, triple sedation with midazolam, fentanyl and propofol for bronchoscopies performed by experienced investigators was not associated with a higher occurrence of complications compared to double sedation with midazolam / fentanyl or midazolam / propofol and led to a reduced consumption of midazolam and propofol. Hence, prospective studies evaluating patient comfort and safety, as well as operating conditions under triple sedation during bronchoscopy might be of interest.

## Supporting information

S1 Table Individual doses of sedative drugs and number of diagnostic interventions for each bronchoscopy included in the analysis. (XLSX)
